# Diagnostic performance analysis for diabetic cardiovascular autonomic neuropathy based on short-term heart rate variability using Bayesian methods: preliminary analysis

**DOI:** 10.1186/s13098-015-0070-z

**Published:** 2015-09-10

**Authors:** Jun Chen, Shuang-Bin Yang, Juanmei Liu, Zi-Hui Tang

**Affiliations:** Department of Endocrinology and Metabolism, Shanghai Tongji Hospital, Tongji University School of Medicine, Rm 1520 Building 6th, No. 389 Xincun Road, Shanghai, 200065 China; Department of Internal Medicine, The People’s Hospital of Mengzi, Honghe, Yunnan China

**Keywords:** Bayesian estimation, Heart rate variability, Diabetic cardiovascular autonomic neuropathy, Without a gold standard

## Abstract

**Objectives:**

This study aimed to estimate the performance of diabetic cardiovascular autonomic neuropathy (DCAN) diagnostic tests in the absence of a gold standard.

**Background:**

The DCAN prevalence is rapidly growing in all populations worldwide. No document has been reported about diagnostic performance for DCAN based on short-term HRV without a gold standard.

**Methods:**

We conducted a cross-sectional study to perform diagnostic test in Chinese diabetic patients. A dataset contained 56 subjects who completed both the short-term HRV test and Ewing’s test. Simultaneous inferences about the population prevalence and the performance of each diagnostic test were possible using the Bayesian approach.

**Results:**

The HRV test had a high sensitivity (0.837 and 0.821 for independence model) and specificity (0.838 and 0.797 for dependence model) to DCAN. In addition, the non-inferiority test rejected the hypothesis that the performance of the HRV test was inferior to that of Ewing’s test (*P* < 0.05). The estimated DCAN prevalence in our study sample was more than 0.400.

**Conclusion:**

Our findings provided evidence that short-term HRV were used for the DCAN diagnostic test with a high sensitivity and specificity.

ClinicalTrial.org ID: NCT02461381

**Electronic supplementary material:**

The online version of this article (doi:10.1186/s13098-015-0070-z) contains supplementary material, which is available to authorized users.

## Background

Diabetic cardiovascular autonomic neuropathy (DCAN) is one of the most overlooked of all serious complications of diabetes [1–3], which encompasses damage to the autonomic nerve fibers that innervate the heart and blood vessels, resulting in abnormalities in heart rate control and vascular dynamics [4]. DCAN is rapidly growing in all populations worldwide, particularly in the developing world [5]. DCAN may carry an increased risk of mortality. Patients with DCAN have an unfavorable cardiovascular risk profile, especially in terms of sudden death, indicating a higher risk of cardiovascular disease [5].

Ewing tests and spectral analysis of spontaneous heart rate variability (HRV) were two widely acceptable methods for cardiovascular autonomic function (CAF) test [6, 7]. Ewing’s test, including five simple noninvasive cardiovascular autonomic reflex tests, had a high sensitivity and specificity for CAF test, [8–11]. However, this test required personnel with specialized skills and was not readily available in general practice [10, 12]. Spectral analysis of HRV had the advantage of quantitatively assessing cardiovascular autonomic activity, and it yields results that were similar to those produced by Ewing’s test [10–13]. Short-term HRV was simple, noninvasive, and reproducible so as to be easily applied in CAF tests in a large-scale sample [11–13]. Our previous study indicated that the short-term HRV indices had a significant negative value correlated with DM, HT, and MS [14]. In general Chinese population, our previous studies evaluated the reference values of short-term HRV, and used these values to create diagnostic criteria for CAN [15]. Although Ewing’s test was acceptable for CAF test in clinical practice, there was no widely accepted a gold standard approach to DCAN diagnosis. Furthermore, we performed a diagnostic study in which Bayesian approaches had been used to estimate diagnostic performance of short-term HRV test for CAN and non-inferior tests provided evidence that this test was similar to Ewing’s test for CAN diagnosis in a general Chinese sample [16–18]. Interestingly, Bayesian statistics had been use to diagnostic performance of tests for diseases in the absence of a gold standard [19, 20]. In general, the Bayesian approach to inference about a generic parameter θ combines prior information about θ with the data to obtain the posterior distribution of θ, p(θ|data). Then, one can use the mean, median, or mode of this posterior distribution as an estimate of θ. Once one can obtain a sample from p(θ|data), a Monte Carlo based estimate of θ can be calculated.

Our previous studies focused on clinical analysis for CAN in general Chinese population, but not in diabetic patients [16]. However, no documents have been reported about short-term HRV test for CAN diagnosis without a gold standard in Chinese diabetic patients. The objective of this study was to estimate diagnostic performance of short-term HRV test for DCAN using the Bayesian approach without a gold standard. Moreover, comparison of diagnostic performance of the two tests for DCAN was analyzed.

## Methods

### Study population

This study is a CAN factor survey carried out in a random sample of the middle-aged Chinese population [14]. Participants that were simultaneously assessed CA function using both the short-term HRV and Ewing’s tests were recruited from department of endocrinology and metabolism and a healthy examination center of Huashan hospital. Survey participants with undiagnosed CAN, aged 30–80 years, were included in this study. More than 100 subjects were invited to a screening visit between 2011 and 2012. Some subjects were excluded from the study to eliminate potential confounding factors that may have influenced their CA function [14]. Briefly, the exclusion criteria were as follows: (1) history or findings of arrhythmia, and hyperthyroidism or hypothyroidism; (2) pregnancy or lactation; and/or (3) serious hepatic or renal dysfunctions. Of these subjects, complete baseline data were obtained for 88 of the participants. Finally, data from 56 dietetic patients have been used to analysis for DCAN diagnostic performance. Written consent was obtained from all patients before the study. This study was approved by the Ethics Committee of the Huashan Hospital, Shanghai, China.

### Measurement

The subjects were interviewed for the documentation of medical histories and medication, history of smoking habits and laboratory assessment of cardiovascular disease risk factors. All study subjects underwent a complete clinical baseline characteristics evaluation after an 8-hour fast, which included: (1) history and physical examination, (2) heart rate and blood pressure, (3) FPG and insulin, and (4) fasting plasma lipids. The FPG was quantified by the glucose oxidase procedure. Serum total cholesterol (TC), HDL cholesterol, TG levels, creatinine (Cr), and uric acid (UA) were measured by an enzymatic method with a chemical analyzer (Hitachi 7600-020, Tokyo, Japan). Low-density lipoprotein (LDL) cholesterol levels were calculated using the Friedewald formula. At the central laboratory in our hospital, the day-to-day and inter-assay coefficients of variation for all analyses were between 1 and 3 %.

### Diagnostic tests

Ewing’s test for the detection of subclinical CAN was carried out as previously described [6]. Briefly, HRV values were analyzed during three maneuvers: deep-breathing (DB), lying-to-standing (LS), and Valsalva (V) tests. The DB test consisted of six deep respiratory cycles in 1 min. The result of the DB test was expressed as the mean value for the ratio of maximal interval between two consecutive R waves on the ECG (RR) during breathing out, over minimal RR during breathing in at each respiratory cycle. The result of the LS test was expressed as the ratio of the longest RR interval (about the 30th beat after standing up) over the shortest RR interval (about the 15th beat). The Valsalva test was performed three consecutive times, and the mean value for the Valsalva ratio (VR) was defined by the longest RR interval after Valsalva release over the shortest RR interval during the active phase of Valsalva. Cardiac parasympathetic neuropathy was considered to be present when at least one test was abnormal according to age. The other two tests investigated BP response to the LS test and to a standard handgrip test. Postural hypotension was assessed by measuring BP after 10 min in the recumbent position and again after 1 min in the standing position. Postural hypotension was defined as a drop in SBP of ≥20 mmHg or in DBP of ≥10 mmHg. The handgrip test consisted of determining the maximal contraction with a dynamometer and then maintaining one-third of the maximal contraction for 5 min. An increase in DBP lower than 10 mmHg was considered to be abnormal. The three tests evaluating HRV are mainly dependent on parasympathetic control, whereas the other two tests evaluating BP response are mainly dependent on sympathetic activity.

The HRV values were measured non-invasively by power spectral analysis. Subjects were studied while awake in the supine position after 20 min of rest. Testing times were from 8:00 to 11:00 in the morning. A type-I FDP-1 HRV noninvasive detecting system was used with software version 2.0 (Department of Biomedical Engineering of Fudan University, Shanghai, China). Electrocardiography, respiratory signals, and beat-to-beat blood pressure were continually and simultaneously recorded for 15 min through an electrosphygmograph transducer (HMX-3C placed on the radial artery of the dominant arm) and an instrument respiration sensor. The short-term HRV analysis was performed for all subjects using a computer-aided examination and evaluation system for spectral analysis to investigate changes in autonomic regulation. The following HRV parameters were measured by frequency domain spectral analysis [10]: total power (TP), lower frequency (LF), normalization LF (LFn), high frequency (HF), and normalization HF (HFn). The TP is the variance of the normal-to-normal interval (NN) over a temporal segment; HF is closely related to vagal activity. The LF/HF ratio was calculated because it is considered to reflect sympatho-vagal balance or sympathetic modulation [12].

### Definition

The HT was defined as a repeatedly elevated blood pressure exceeding 140 over 90 mmHg—a systolic pressure above 140 or a diastolic pressure above 90. The DM was diagnosed by demonstrating any one of the following: (1) FPG level ≥7.0 mol/l; (2) plasma glucose ≥11.1 mol/l 2 h after a 75 g oral glucose tolerance test (OGTT); (3) symptoms of hyperglycemia and casual plasma glucose ≥11.1 mol/l and (4) glycated hemoglobin (HbA1C) ≥6.5 %. The BMI was calculated by weight in kilograms divided by the square of height in meters. Metabolic syndrome was diagnosed in individuals who met three or more criteria of the updated National Cholesterol Education Program/Adult Treatment Panel III (WHO Western Pacific Region obesity criteria) [21]. In this study, CAN was diagnosed based on at least two abnormal CA reflex test results (based on Ewing’s test model or HRV test model) [11, 15]. DCAN was diagnosed in diabetic patients with CAN.

### Statistical analysis

The Kolmogorov–Smirnov (K–S) test was used to determine whether continuous variables followed a normal distribution. Variables that were not normally distributed were log-transformed to approximate normal distribution for analysis. The results are expressed as the mean ± standard deviation or median, unless otherwise stated. Pearson and Spearman analytical methods were employed for correlation analysis of two variables. In general, we report skewed data in tables with 2.5th and 97.5th percentiles or median, however, in this study we reported skewed data involved in indices of HRV with mean and SD format because HRV parameters often were presented with the format in other studies.

We used a Bayesian latent class model to estimate the sensitivity and specificity of the HRV test and/or Ewing’s test for DCAN in the absence of a gold standard, as described by Branscum et al. [22]. The latent class analysis allows the characterization of a discrete latent class (here, the true disease status) by discrete observed variables. In this model, both tests are equally considered as imperfect. There are unknown parameters about which inference must be made: the DCAN population prevalence, and the sensitivity and specificity of each of the two tests. The Bayesian approach can simultaneously estimate all five unknown parameters. These methods proceed in two steps: first, a prior distribution summarizes the available pre-experimental information about the parameters. Subsequently, the prior distribution is updated via Bayes’ theorem to a posterior distribution, using the data and the usual multinomial likelihood function. Marginal posterior densities can be derived for each parameter by integration, from which 95 % marginal posterior credible intervals can be calculated. Since the integration here is analytically intractable, the Gibbs sampler, a Monte Carlo approach to calculating marginal densities, is employed. The above methods allow for simultaneous inferences to be made for all unknown parameters, which take full advantage of all the information contained in the data, as well as formally incorporate prior information, when available (see Additional file 1). A minimum sample size estimation for this diagnostic performance analysis was 50 subjects by using sample size estimation formula: N = Z^2^Sen(1 − Sen)/deta^2^ + Z^2^Spe(1 − Spe)/deta^2^; here, Z derived form alpha level (0.05 in this study), Sen (sensitivity) and Spe (specificity) were set to 0.85, respectively; deta was set to 0.10–0.20 in this study. Data were analyzed using SPSS16.0 (USA) and WinBUGS.14 for the Bayesian analysis.

## Results

The baseline characteristics of the 56 subjects are listed in Table 1. The entire sample included 24 males and 32 females (mean age, 59.21 ± 8.22 years). The majority of subjects had never smoked (75.37 %), and the prevalence of HT and MS were 64.29 % and 57.14 % in the entire sample, respectively. The most of demographic parameters, parameters of blood glucose, lipid profiles, and medical histories of diabetic patients without CAN were similar to those of patients with DCAN (P >0.05 for all). However, there were significantly greater levels of SBP and FPG in patients with DCAN as compared with those of diabetic patients without CAN, respectively (P = 0.013 for SBP and P = 0.025 for FPG). Furthermore, patients with DCAN have longer duration of DM and PH compared to those of diabetic patients without CAN, respectively (P = 0.026 for DM duration and P = 0.001 for PH duration).

**Table 1:** Baseline characteristics of subject

Variable	Total sample	non-DCAN	DCAN	P value
Demographic information				
N	56	22	34	
Age (years)	59.21 ± 8.22	59.82 ± 9.7	58.91 ± 7.64	0.769
Gender male (%)	24 (42.86 %)	10 (45.45 %)	19 (55.88 %)	0.340
Height (cm)	164.32 ± 6.74	163.82 ± 6.69	164.57 ± 6.89	0.767
Weight (kg)	63.88 ± 9.61	63.91 ± 10.29	63.87 ± 9.5	0.991
WC (cm)	84.29 ± 6.73	82.82 ± 6.26	85 ± 6.97	0.385
SBP (mmHg)	125.85 ± 10.62	120.31 ± 10.62	129.26 ± 11.22	0.013
DBP (mmHg)	81.23 ± 9.59	79.26 ± 8.79	82.68 ± 9.39	0.156
Laboratory assays				
FPG (mmol/L)	6.84 ± 1.93	6.56 ± 1.78	7.02 ± 2.01	0.025
TC (mmol/L)	5.35 ± 1.05	5.2 ± 1.01	5.45 ± 1.08	0.365
TG (mmol/L)	1.86 ± 1.07	1.78 ± 1.02	1.91 ± 1.09	0.452
HDL (mmol/L)	1.30 ± 0.30	1.27 ± 0.28	1.32 ± 0.31	0.619
LDL (mmol/L)	3.14 ± 0.79	3 ± 0.71	3.23 ± 0.89	0.767
UA (μmol/L)	292.34 ± 82.65	276.62 ± 79.65	302.51 ± 85.65	0.477
HRV measurement				
HR (bpm)	73.62 ± 11.09	71.3 ± 7.98	74.81 ± 12.31	0.265
TP (ms^2^)	731.49 ± 895.49	832.61 ± 814.17	679.56 ± 941.02	0.550
LF (ms^2^)	144.85 ± 245.48	155.49 ± 228.32	139.38 ± 256.73	0.819
LFnu	16.82 ± 9.81	17.13 ± 7.25	16.66 ± 10.99	0.867
HF (ms^2^)	191.28 ± 460.81	204.79 ± 283.98	184.33 ± 532.87	0.877
HFnu	20.08 ± 15.01	20.66 ± 14.24	19.78 ± 15.57	0.837
LF/HF	1.33 ± 1.15	1.46 ± 1.33	1.26 ± 1.05	0.554
Medical history				
Smoking yes (%)	15 (41.67 %)	5 (22.73 %)	10 (29.41 %)	0.956
HT yes (%)	36 (64.29 %)	13 (59.09 %)	23 (67.65 %)	0.471
MS yes (%)	32 (57.14 %)	11 (50.00 %)	21 (61.76 %)	0.856
DM duration	7.3 ± 1.45	6.55 ± 1.60	7.66 ± 1.28	0.026
PH duration	4.4 ± 1.38	1.86 ± 1.03	5.61 ± 1.34	0.001

*WC* waist circumference, *SBP* systolic blood pressure, *DBP* diastolic blood pressure, *FPG* fasting plasma glucose, *TC* serum total cholesterol, *TG* triglyceride, *UA* uric acid, *HDL* high-density lipoprotein cholesterol, *LDL* low density lipoprotein cholesterol, *HR* heart rate, *TP* total power of variance, *LF* low frequency, *HF* high frequency, *MS* metabolic syndrome, *HT* hypertension, *DM* diabetes

Of the 56 subjects in the external dataset, 27 and 34 subjects were diagnosed with CAN using the HRV test alone and Ewing’s test alone, respectively (Table 2). Using both tests, 21 subjects were diagnosed with CAN, while 16 subjects were diagnosed free of CAN.

**Table 2:** Results of short-term HRV and Ewing’s’ test for cardiovascular autonomic neuropathy in 56 subjects

Model	Ewing’s’ test			Total
		+	−	
HRV test	+	21	6	27
	−	13	16	29
Total		34	22	56

### Prior distributions of Bayesian analysis

Prior distributions can be estimated based on a review of the literature and/or expert opinion in the absence of data. Published evaluations of Ewing’s test indicated a good sensitivity (0.7–1.0) and specificity (0.7–1.0), which has a beta distribution with parameters (α, β) [10–12, 23]. Previous studies demonstrated that performances of HRV to assess CA activity were similar to those of Ewing’s test [12, 24, 25]. We formed a hypothesis for the short-term HRV test with a sensitivity and specificity of a beta distribution between 0.7 and 1.0, respectively. Finally, the prior distribution of prevalence was considered beta between 0.1 and 0.5 [10, 12, 26]. The same parameters of prior distribution for the HRV test alone were estimated in the total sample, and in the DM, HT, and MS patients. The two tests used here relied on the analysis of HRV attributes. As recommended by Dendukuri et al. [27], in the main analysis, the tests were also considered a conditionally independent model. The particular beta prior density for each test parameter was selected by matching the center of the range with the mean of the beta distribution, given by α/(α + β), and matching the variance of the beta distribution, given by the square root of (αβ)/((α + β)^2^(α + β+1)) with one quarter of the total range.

### Bayesian estimation of diagnostic test

When both tests were combined with the conditional independence model, the median posterior DCAN prevalence was 0.429 (95 % CI, 0.278–0.590, Table 3), and the median posterior sensitivity and specificity of the HRV test were 0.837 (95 % CI, 0.681–0.948) and 0.838 (95 % CI, 0.696–0.941), respectively. There was an apparently higher sensitivity (0.878) but a lower specificity (0.745) of Ewing’s test, compared with those of the HRV test. When both tests were combined with the conditional dependence model, the median posterior DCAN prevalence was 0.403 (95 % CI, 0.222–0.591), and the median posterior sensitivity and specificity of the HRV test were 0.821 (95 % CI, 0.626–0.950) and 0.797 (95 % CI, 0.636–0.927), respectively. There were modest correlations between the HRV test and Ewing’s test (*ρ*_P_ = 0.335 and *ρ*_N_ = 0.344).

**Table 3:** Marginal prior and posterior medians and lower and upper limits of the posterior equally tailed 95 % credible interval for parameters of diagnostic tests

Variables	Prior information	Both tests combined (independence)		Both tests combined (dependent)	
		HRV test	Ewing’s test	HRV test	Ewing’s test
Prevalence	0.300 (0.120–0.480)	0.429 (0.278–0.59)		0.403 (0.222–0.591)	
Sensitivity	0.850 (0.720–0.980)	0.837 (0.681–0.948)	0.878 (0.748–0.962)	0.821 (0.626–0.95)	0.852 (0.698–0.956)
Specificity	0.850 (0.720–0.980)	0.837 (0.696–0.941)	0.745 (0.591–0.891)	0.797 (0.636–0.927)	0.695 (0.529–0.888)
Accuracy	0.850 (0.720–0.980)	0.835 (0.727–0.919)	0.810 (0.705–0.9)	0.807 (0.666–0.911)	0.774 (0.647–0.894)
PPV	0.708 (0.260–0.978)	0.796 (0.582–0.934)	0.720 (0.497–0.902)	0.733 (0.451–0.916)	0.651 (0.375–0.901)
NPV	0.923 (0.950–0.982)	0.874 (0.69–0.967)	0.892 (0.743–0.97)	0.873 (0.639–0.972)	0.876 (0.71–0.970)
ρ_P_				0.335 (0.017–0.838)	
ρ_N_				0.344 (0.016–0.838)	

Date represent to be mean and 95 % CI, *PPV* positive predictive value, *NPV* negative predictive value, *ρ*_*P*_ correlation coefficients of sensitivities of two tests, *ρ*_*N*_ correlation coefficients of specificities of two tests

### Non-inferiority analysis for diagnostic parameters

Similar parameters of the HRV for DCAN were found (Table 3). Generally, the median posterior sensitivities and specificities of the HRV test were over 80 % in all models. Higher sensitivities and lower specificities of Ewing’s test were found in all models, compared with those of the HRV test. The posterior Youden indices of the HRV test were higher than those of Ewing’s test in all models. In combined tests, we compared the parameters (mean sensitivity, mean specificity, accuracy, PPV and NPV) for performance of both diagnostic tests by using a non-inferiority test that rejected the hypothesis that the diagnostic performance of the HRV test was inferior to that of Ewing’s test (*P* < 0.05 for all parameters in two models, Table 4), suggesting that the performance of short-term HRV test was similar to Ewing’s test in diagnosis of DCAN.

**Table 4:** Comparison of performance of HRV test and Ewing’s test in combined both tests

Parameter	Both tests combined (independence)					Both tests combined (dependence)				
	HRV test		Ewing’s test		*P* value	HRV test		Ewing’s test		*P* value
	Mean	SE	Mean	SE		Mean	SE	Mean	SE	
Sensitivity	0.830	0.070	0.872	0.056	0.035	0.812	0.086	0.845	0.066	0.086
Specificity	0.833	0.063	0.744	0.077	0.000	0.792	0.075	0.700	0.091	0.006
Accuracy	0.832	0.049	0.808	0.049	0.001	0.802	0.063	0.772	0.062	0.011
PPV	0.786	0.091	0.715	0.104	0.002	0.720	0.119	0.649	0.136	0.016
NPV	0.862	0.072	0.882	0.059	0.005	0.853	0.089	0.866	0.068	0.019

Non-inferiority test to test the hypothesis of performance of HRV test inferior to Ewing’s test; delta >0 is the non-inferiority margin of clinical interest, which is set as quarter of value of parameter of Ewing’s test in this study. *PPV* positive predictive value, *NPV* negative predictive value

## Discussion

A cross-sectional study was conducted to evaluate diagnostic performance of short-term HRV for DCAN in Chinese diabetic patients. Short-term HRV was a simple, noninvasive, and reproducible test for CAF evaluation and was more suitable for clinical application in CAF test. In this study, it is very important for us to understand that the HRV test and Ewing’s test are similar diagnostic tests. Additionally, we paid attention on no widely accepted a gold standard diagnostic criteria for DCAN in clinical practice, and Bayesian analysis to be applied for estimation of diagnostic parameters in the absence of a gold standard. More importantly, it is the first study, using Bayesian approaches in the absence of a gold standard, to estimate the diagnostic performance of short-term HRV for DCAN in Chinese diabetic patients.

An interesting finding is that the diagnostic test for DCAN based on the short-term HRV showed a high sensitivity and specificity. In our study sample, the estimated median sensitivities and specificities of the HRV test for DCAN were up to 0.800 in all models. The median sensitivities was 0.837 and 0.821 in independence and dependence models, respectively. Specially, the high median specificities (0.878 and 0.852 for both model, respectively) was reported, which was important for diagnosis of DCAN in clinical practice. Our previous study conducted Bayesian analysis for CAN in diabetic subgroup under HRV alone model to show this test having high sensitivity (0.843) and specificity (0.866). Under both tests combined model, high sensitivities (0.800-0.822) and specificities (0.812–0.852) were also reported for this test to diagnose CAN in general Chinese population. In this study, we could find that the mean values of short-term HRV indices were higher in patients without DCAN as compared to patients with DCAN. However, there were no differences in HRV indices between the two groups. These results were not consistent with previous findings in other literature. This was partly because the description statistics of raw data of HRV indices showed to those parameters with large variances in our study. Additionally, this may be our study with a moderate sample size. However, we set the reference value using the 5th percentiles and combined at least two indices value for diagnostic criteria of CAN. The diagnostic criteria have enough power to determine outcomes, although there were no differences in HRV indices between the two groups. Moreover, the results of Bayesian analysis supported the fact that short-term HRV for diagnosis of CAN have high sensitivity and specificity. More importantly, future studies with design well will test the influence of heart rate. Our results provided evidence that the HRV test is an efficient test for diagnosis of DCAN in clinical practice.

It was very important to evaluate the performance of the HRV test in both tests combined with the independence and dependence models using the Bayesian approach. The combined tests allowed for sharper inferences to be drawn [28]. Studies were generally performed to evaluate the performance of new diagnostic tests using Ewing’s as a reference, however, the test was actually not a gold standard. Using the Bayesian approach, in the absence of a gold standard, simultaneous inferences about the performance of each diagnostic test are possible. Additionally, Bayesian estimation of the parameters of a diagnostic test needs prior information. In this study, precise and accurate posterior parameters should be derived from appropriate prior parameters. This is more suitable for clinical research because it is easier for experts with relevant clinical experience to estimate prior parameters accurately.

A non-inferiority test indicated that the most of parameters was not inferior to that of Ewing’s test, except sensitivity parameter under both test combined with dependence model. These findings provided evidence that the HRV test was not inferior to Ewing’s test for DCAN diagnosis. To our knowledge, this is the first study to have reported that in the absence of a gold standard, short-term HRV test for DCAN diagnosis had high sensitivity and specificity. Previously, Ewing’s test was a widely acceptable test for CAF evolution to be applied in DCAN diagnosis. Therefore, in this study, our finding is of importance to the clinical practice of DCAN diagnosis in diabetic patients.

In our study sample, under both tests models, estimated median prevalence of DCAN was more than 0.400. In our previous study, under HRV alone model, its prevalence was estimated at 0.292 in diabetic patients. Laitinen et al. [29] reported that the prevalence of parasympathetic dysfunction was 25 % in subjects with central obesity in persons with impaired glucose tolerance. Additionally, the estimated CAN prevalence in diabetic patients was found to be 20–50 % in other studies [10, 11], indicating that our result was consistent with these studies. In our study sample, under the HRV test alone, CAN was estimated at 0.149 in the general population, suggesting that CAN was more frequent in diabetic patients. Our findings supported evidence that CAN has become a serious public problem in China. A higher prevalence of this disease was found in special subgroups.

Several limitations of this study warrant comment. This study does not cover age groups other than 30–80 years. Additionally, a cross-sectional study for the determination of diagnostic test requires a larger sample size and more geographic representations. Furthermore, our findings need to be verified by future multiple centers studies and/or follow-up studies. Finally, it is important to mention that our study was performed on Chinese individuals, and our findings may not be relevant to people of other ethnicities.

In conclusion, this study provided evidence that short-term HRV test has a high sensitivity and specificity for DCAN diagnosis, and that the HRV test was not inferior to the traditional Ewing’s test for DCAN. The estimated DCAN prevalence was high in diabetic patients. DCAN requires strategies to prevent and treat the disease (Additional file 1).

## Strengths and limitations of this study

1. The first study to evaluate performance of diabetic cardiovascular autonomic neuropathy diagnosis based on short-term heart rate variability test by using Bayesian analysis without a gold standard
2. Comparison of performance of diagnosis based on short-term heart rate variability test and Ewing’s test by using non-inferiority test
3. Data interpretation and analysis be applied in Chinese population, but lack of evidence to applied in other areas or ethnics

## Additional file

Additional file 1. Supplementary appendix for diagnostic performance analysis for diabetic cardiovascular autonomic neuropathy based on short-term heart rate variability using Bayesian methods: preliminary analysis

## Abbreviations

BP: blood pressure
BMI: body mass index
DCAN: diabetic cardiovascular autonomic neuropathy
CI: credible intervals
Cr: creatinine
DM: diabetes
FPG: fasting plasma glucose
HDL: High-density lipoprotein cholesterol
HF: high frequency
HRV: heart rate variability
LDL: low-density lipoprotein cholesterol
LF: low frequency
MS: metabolic syndrome
K–S: Kolmogorov–Smirnov
OGTT: oral glucose tolerance test
PBG: postprandial blood glucose
HT: hypertension
TC: serum total cholesterol
TG: triglyceride
TP: total power
WC: waist circumference
UA: uric acid
