# Erratum to: Diagnostic performance analysis for diabetic cardiovascular autonomic neuropathy based on short-term heart rate variability using Bayesian methods: preliminary analysis

**DOI:** 10.1186/s13098-017-0213-5

**Published:** 2017-02-16

**Authors:** Jun Chen, Shuang-Bin Yang, Juanmei Liu, Zi-Hui Tang

**Affiliations:** 1 Department of Endocrinology and Metabolism, Shanghai Tongji Hospital, Tongji University School of Medicine, Rm 1520 Building 6th, No. 389 Xincun Road, Shanghai, 200065 China; 2 Department of Internal Medicine, The People’s Hospital of Mengzi, Honghe, Yunnan China

## Erratum to: Diabetol Metab Syndr (2015) 7:74 DOI 10.1186/s13098-015-0070-z

We acknowledge that in the published work [1], text in multiple sections of the article overlaps with our previous publication, Tang et al. 2014 BMJ Open [2]. We would like to further clarify the relationship between these manuscripts. In 2013, we had collected and analyzed the data from general population to estimate the diagnostic performance of short-term heart rate variability for cardiovascular autonomic neuropathy by using Bayesian approaches, these data were published in BMJ Open article [2]. In 2014, we collected and analyzed the data from diabetic patients to estimate the performance of diabetic cardiovascular autonomic neuropathy by using similar Bayesian approaches, these data were included in the manuscript published in Diabetology & Metabolic Syndrome [1]. We would like to clarify that we consider that the aims, dataset, outcomes, and highlights of the two papers are different and both results are very important for clinicians. We apologize for any inconvenience caused.
