# Supplementary material for: Diagnostic performance analysis for diabetic cardiovascular autonomic neuropathy based on short-term heart rate variability using Bayesian methods: preliminary analysis
Source: Diabetol Metab Syndr. 2015 Sep 10;7:74. doi: 10.1186/s13098-015-0070-z (PMC4566203; doi:10.1186/s13098-015-0070-z)
Supplement: Additional file 1. — Supplementary appendix for diagnostic performance analysis for diabetic cardiovascular autonomic neuropathy based on short-term heart rate variability using Bayesian methods: preliminary analysis [file 13098_2015_70_MOESM1_ESM.pdf]

Supplementary Appendix

This document has been provided by the authors to give reviewers additional information about their work.

Supplement to: Diagnostic performance analysis for diabetic cardiovascular autonomic neuropathy based on short-term heart rate variability using Bayesian methods: Preliminary analysis.

Outline of Contents

1. Overview 3
2. Prior distribution 4
3. One diagnostic test 5
4. Two diagnostic tests (conditional independence model) 8
5. Two diagnostic tests (conditional dependence model) 11
6. WinBUGS program code for two independence diagnostic test 14
Reference: 15

1. Overview

W We used a Bayesian latent class model to estimate the sensitivity and specificity of HRV test or/and Ewing's test for CAN in the absence of a gold standard, as described by Branscum et al. [1]. The latent class analysis allows the characterization of a discrete latent class (here the true disease status) by discrete observed variables. In this model, both tests are equally considered as imperfect. There are unknown parameters about which inference must be made: the CAN population prevalence, and the sensitivity and specificity of each of the two tests. Bayesian approach can simultaneously estimate all five unknown parameters. These methods proceeds in two steps: first, a prior distribution summarizes the available pre-experimental information about the parameters. Subsequently, the prior distribution is updated via Bayes Theorem to a posterior distribution, using the data and the usual multinomial likelihood function. Marginal posterior densities can be derived for each parameter by integration, from which 95% marginal posterior credible intervals can be calculated. Since the integration here is analytically intractable, the Gibbs Sampler, a Monte Carlo approach to calculating marginal densities, is employed. The above methods allow for simultaneous inferences to be made for all unknown parameters, which takes full advantage of all the information contained in the data, as well as formally incorporating prior information, when available. Data were analyzed using SPSS16.0 (USA) and WinBUGS 14 for the Bayesian analysis.

2. Prior distribution

Prior distributions can be estimated based on a review of the literature and/or expert opinion in the absence of data. Published evaluations of the Ewing's test indicated a good sensitivity (0.7 to 1.0) and specificity (0.7 to 1.0), which has a beta distribution with parameters (α, β) [2,3,4,5]. Previous studies demonstrated that performances of HRV to assess CA activity are similar to those of Ewing's test [4,6,7]. We made a hypothesis for the short-term HRV test with sensitivity and specificity of a beta distribution between 0.7 and 1.0, respectively. Finally, the prior distribution of prevalence was considered beta between 0.1 and 0.5 [3,4,8]. The same parameters of prior distribution for HRV test alone were estimated in total sample, DM, HT and MS patients. The two tests used here rely on analysis of HRV attributes. As recommended by Dendukuri et al. [9], in the main analysis the tests were also considered conditionally independent model. The particular beta prior density for each test parameter was selected by matching the center of the range with the mean of the beta distribution, given by $\alpha/(\alpha+\beta)$, and matching the variance of the beta distribution, given by square root of $(\alpha\beta)/((\alpha+\beta)^2(\alpha+\beta+1))$ with one quarter of the total range.

3. One diagnostic test

Let A and B be the observed number of positive and negative test results, respectively, in the sample of A + B = N subjects (Table a1). Let Y_1 and Y_2 be the information that is missing when there is no gold standard, that is, the number of true positive test results out of A and B, respectively. Thus, Y_1 is the number of true positives, and Y_2 is the number of false negatives. Such missing information has been termed "latent data".

Table a1: Observed and latent data in the case of one diagnostic test In the absence of a gold standard, presented in a 2 x 2 table

		True		Total
		Positive	Negative	
Test	Positive	Y_1	$A-Y_1$	A
	Negative	Y_2	$B-Y_2$	B
	Total	Y_1+Y_2	$N-Y_1-Y_2$	N

The likelihood function of the observed and latent data is given by

$$L(A, B, Y_1, Y_2 | \pi, Sen, Spe) = [\pi Sen]^{Y_1}[\pi(1-Sen)]^{Y_2}[(1-\pi)(1-Spe)]^{A-Y_1}[(1-\pi)Spe]^{B-Y_2} .$$

Prior information in the form of a beta density will be assumed. A random variable ($0 \leq \theta \leq 1$) has a beta distribution with parameters (α, β) if it has a probability density given by

$$f(\theta;\alpha,\beta) = \frac{1}{B(\alpha,\beta)}\theta^{\alpha-1}(1-\theta)^{\beta-1}$$

, where $B(\alpha, \beta)$, the beta function evaluated at (α, β), is the normalizing constant. This family of distributions was selected since its region of positive density, from 0 to 1, matches the range of all parameters of interest in this study. In addition, it also has the advantage of being the conjugate prior distribution for the binomial likelihood, a property that simplifies the derivation of the posterior distributions. Let (α_π, β_π), (α_{Sen}, β_{Sen}), and (α_{Spe}, β_{Spe}) represent the prior beta parameters for π, Sen and Spe, respectively. Since the joint posterior distribution is proportional to the product of the likelihood function and the prior distribution.

Inference is possible using a Gibbs sampler algorithm. The basic idea is as follows.

Conditional on knowing the exact values of the prevalence and all diagnostic test parameters, it is possible to derive posterior distributions of the latent data Y_1 and Y_2. Conversely, if Y_1 and Y_2 are known, then deriving posterior distributions of the prevalence and diagnostic test parameters given the prior distributions requires only a straightforward application of Bayes' theorem. An algorithm that alternates between these two steps can thus be devised, similar in spirit to the expectation maximization algorithm that is commonly used in latent class analysis. The Gibbs sampler algorithm provides random samples from the marginal posterior densities of each parameter of interest. These random samples can then be used to reconstruct the marginal posterior densities, or summaries of these densities, such as their means, medians, or standard deviations, as well as probability interval summaries.

Implementation of the Gibbs sampler requires the specification of the full conditional distributions of the parameters, i.e., the conditional distributions of each parameter given the values of all of the other parameters. It is straightforward to show from likelihood function that the following conditional distributions must hold:

$$Y_1 \mid A, \pi, Sen, Spe \sim Binomial\left(A, \frac{\pi Sen}{\pi Sen + (1-\pi)(1-Spe)}\right) \quad \text{(app1.1)}$$

$$Y_2 \mid B, \pi, Sen, Spe \sim Binomial\left(B, \frac{\pi(1-Sen)}{\pi(1-Sen) + (1-\pi)Spe}\right) \quad \text{(app1.2)}$$

$$\pi \mid A, B, Y_1, Y_2, \alpha_\pi, \beta_\pi \sim Beta(Y_1+Y_2+\alpha_\pi, A+B-Y_1-Y_2+\beta_\pi) \quad \text{(app 1.3)}$$

$$Sen \mid Y_1, Y_2, \alpha_{Sen}, \beta_{Sen} \sim Beta(Y_1+\alpha_{Sen}, Y_2+\beta_{Sen}) \quad \text{(app 1.4)}$$

and

$$Spe \mid A, B, Y_1, Y_2, \alpha_{Spe}, \beta_{Spe} \sim Beta(B-Y_2+\alpha_{Spe}, A-Y_1+\beta_{Spe}) \quad \text{(app 1.5)}$$

The Gibbs sampler operates as follows. Arbitrary starting values are chosen for each parameter. A sample of size m is then drawn from each full conditional distribution, in turn. The sampled values from the previous iterations are used in the conditional distributions for subsequent iterations. A cycle of the algorithm is completed when all conditional distributions have been sampled at least once. The entire cycle is repeated a large number of times. The random samples thus generated for each parameter can be regarded as a random sample from the correct posterior marginal distribution. For the above model, Y_1 and Y_2 are

generated from expressions app 1.1 and app1.2, respectively, given the starting values of the other parameters. Then, π is generated from equation app1.3 conditional on the Y_1 and Y_2 variates just sampled. Drawing Sen and Spe from densities given in expressions app1.4 and app1.5, respectively, using the same values of Y_1 and Y_2 completes the first cycle. Positive and negative predictive values can be computed after each cycle from Y_1/A and $(b-Y_2)/B$, respectively. The random samples generated by repeating the above cycle the desired number of times are then used to reconstruct the marginal posterior densities of each parameter and to find credible sets, marginal posterior means or medians, or other inferences.

4. Two diagnostic tests (conditional independence model)

The methods of the previous section can be extended to the situation where results of two diagnostic tests for the same disease are available on a randomly selected sample of subjects, where neither test can be considered a gold standard. There are unknown five parameters about which inference must be made: the population prevalence of CA dysfunction (π), and the sensitivity (S_1) and specificity (C_1) of the test1, and sensitivity (S_2) and specificity (C_2) of the Test2. Let U_1 be the observed number of positive test1 and test2 results, and U_2 be the observed number of positive test1 and negative test2 results, and U_3 the observed number of negative test1 and positive test2 results, and U_4 be the observed number of negative test1 and test2 results, in the sample of $U_1+U_2+U_3+U_4 = N$ subjects (Table a2).

Table a2: Observed data from two diagnostic tests, In the absence of a gold standard

		Test2		Total
		Positive	Negative	
Test1	Positive	U_1	U_2	U_1+U_2
	Negative	U_3	U_4	U_3+U_4
	Total	U_1+U_3	U_2+U_4	N

Let the unobserved latent data Y_1, Y_2, Y_3, and Y_4 represent the number of true positive subjects out of the observed cell values U_1, U_2, U_3 and U_4, respectively. Since any subject, whether truly possessing the disease in question or not, can test positively or negatively on each test, there are eight possible combinations.

Table a3: Likelihood contributions of all possible combinations of observed and latent data for the case of two independence diagnostic tests

No. of sub	Truth	Test1 result	Test2 result	Likelihood Contribution
Y_1	Positive	Positive	Positive	$\pi S_1 S_2$
Y_2	Positive	Positive	Negative	$\pi S_1(1-S_2)$
Y_3	Positive	Negative	Positive	$\pi(1-S_1)S_2$
Y_4	Positive	Negative	Negative	$\pi(1-S_1)(1-S_2)$
U_1-Y_1	Negative	Positive	Positive	$(1-\pi)(1-C_1)(1-C_2)$
U_2-Y_2	Negative	Positive	Negative	$(1-\pi)(1-C_1)C_2$
U_3-Y_3	Negative	Negative	Positive	$(1-\pi)C_1(1-C_2)$
U_4-Y_4	Negative	Negative	Negative	$(1-\pi)C_1C_2$

Note: The likelihood is proportional to the product of each entry In the last column of the table raised to the power of the corresponding entry In the first column of the table.

The likelihood function of the observed and latent data is given by (Table a3):

$$L(U_1,U_2,U_3,U_4,Y_1,Y_2,Y_3,Y_4|\pi,S_1,C_1,S_2,C_2)$$
$$=[\pi S_1S_2]^{Y_1}[\pi S_1(1-S_2)]^{Y_2}[\pi(1-S_1)S_2]^{Y_3}[\pi(1-S_1)(1-S_2)]^{Y_4}$$
$$[(1-\pi)(1-C_1)(1-C_2)]^{U_1-Y_1}[(1-\pi)(1-C_1)C_2]^{U_2-Y_2}[(1-\pi)C_1(1-C_2)]^{U_3-Y_3}[(1-\pi)C_1C_2]^{U_4-Y_4}$$

We used standard distributional families to represent our prior information. The choice of distributions discussed below is not unique and they may be replaced by other suitable densities, as needed. The prevalence is assumed to follow a beta prior distribution with parameters α and β, $\pi \sim \text{beta}(\alpha_\pi, \beta_\pi)$. The sensitivities and specificities are also assumed to have beta prior densities such that $S_j \sim \text{beta}(\alpha_{Sj}, \beta_{Sj})$, and $C_j \sim \text{beta}(\alpha_{Cj}, \beta_{Cj})$, j=1,2. The Gibbs sampler can again be used to construct the marginal posterior densities of all parameters of interest. For two independence diagnostic tests, the full conditional distributions are as follows:

$$Y_1|U_1,\pi,S_1,C_1,S_2,C_2 \sim Binomial(U_1,\frac{\pi S_1S_2}{\pi S_1S_2+(1-\pi)(1-C_1)(1-C_2)}) \quad \text{(app2.1)}$$

$$Y_2|U_2,\pi,S_1,C_1,S_2,C_2 \sim Binomial(U_2,\frac{\pi S_1(1-S_2)}{\pi S_1(1-S_2)+(1-\pi)(1-C_1)C_2}) \quad \text{(app2.2)}$$

$$Y_3|U_3,\pi,S_1,C_1,S_2,C_2 \sim Binomial(U_3,\frac{\pi(1-S_1)S_2}{\pi(1-S_1)S_2+(1-\pi)C_1(1-C_2)}) \quad \text{(app2.3)}$$

$$Y_4 | U_4, \pi, S_1, C_1, S_2, C_2 \sim Binomial(U_4, \frac{\pi (1-S_1)(1-S_2)}{\pi (1-S_1)(1-S_2) + (1-\pi) C_1 C_2}) \quad \text{(app2.4)}$$

$$\pi | U_1, U_2, U_3, U_4, Y_1, Y_2, Y_3, Y_4, \alpha_\pi, \beta_\pi, \sim Beta(Y_1+Y_2+Y_3+Y_4+\alpha_\pi, N-(Y_1+Y_2+Y_3+Y_4)+\beta_\pi) \quad \text{(app2.5)}$$

$$S_1 | Y_1, Y_2, Y_3, Y_4, \alpha_{S1}, \beta_{S1} \sim Beta(Y_1+Y_2+\alpha_{S1}, Y_3+Y_4+\beta_{S1}) \quad \text{(app2.6)}$$

$$S_2 | Y_1, Y_2, Y_3, Y_4, \alpha_{S2}, \beta_{S2} \sim Beta(Y_1+Y_3+\alpha_{S2}, Y_2+Y_4+\beta_{S2}) \quad \text{(app2.7)}$$

$$C_1 | U_1, U_2, U_3, U_4, Y_1, Y_2, Y_3, Y_4, \alpha_{C1}, \beta_{C1} \sim Beta(U_3+U_4-(Y_3-Y_4)+\alpha_{C1}, U_1+U_2-(Y_1+Y_2)+\beta_{C1}) \quad \text{(app2.8)}$$

$$C_2 | U_1, U_2, U_3, U_4, Y_1, Y_2, Y_3, Y_4, \alpha_{C2}, \beta_{C2} \sim Beta(U_2+U_4-(Y_2-Y_4)+\alpha_{C2}, U_1+U_3-(Y_1+Y_3)+\beta_{C2}) \quad \text{(app2.9)}$$

5. Two diagnostic tests (conditional dependence model)

Assume that we have results from two different dichotomous tests T_j, j=1,2, from a sample of N subjects such that a positive result on the jth test is denoted by T_j=1 and a negative result by T_j=0. Let *D* denote the latent true disease status such that *D*=1 among diseased subjects and *D*=0 among nondiseased subjects. To model the conditional dependence between two diagnostic tests recommended by Dendukuri et al., the conditional dependence between tests may be estimated using a measure such as the covariance between tests within each disease class. We denote the covariance between the two tests among the diseased and nodiseased subjects as cov_s and cov_c, respectively. Here, $cov_s=P(T_1=1, T_2=1|D=1)-S_1S_2$, and $cov_c= P(T_1=0, T_2=0|D=0)-C_1C_2$.

Table a4: Likelihood contributions of all possible combinations of observed and latent data for the case of two dependence diagnostic tests

No. of sub	Truth	Test1 result	Test2 result	Likelihood Contribution
Y_1	Positive	Positive	Positive	$\pi(S_1S_2+cov_s)$
Y_2	Positive	Positive	Negative	$\pi(S_1(1-S_2)-cov_s)$
Y_3	Positive	Negative	Positive	$\pi((1-S_1)S_2-cov_s)$
Y_4	Positive	Negative	Negative	$\pi((1-S_1)(1-S_2) +cov_s)$
U_1-Y_1	Negative	Positive	Positive	$(1-\pi)((1-C_1)(1-C_2) +cov_c)$
U_2-Y_2	Negative	Positive	Negative	$(1-\pi)((1-C_1)C_2-cov_c)$
U_3-Y_3	Negative	Negative	Positive	$(1-\pi)(C_1(1-C_2) -cov_c)$
U_4-Y_4	Negative	Negative	Negative	$(1-\pi)(C_1C_2+cov_c)$

Note: The likelihood is proportional to the product of each entry In the last column of the table raised to the power of the corresponding entry In the first column of the table.

The likelihood function of the observed and latent data is given by (Table a4):

$$L(U_1,U_2,U_3,U_4,Y_1,Y_2,Y_3,Y_4|\pi,S_1,C_1,S_2,C_2,\mathrm{cov}_s,\mathrm{cov}_c)$$
$$=[\pi(S_1S_2+\mathrm{cov}_s)]^{Y_1}[\pi(S_1(1-S_2)-\mathrm{cov}_s)]^{Y_2}[\pi((1-S_1)S_2-\mathrm{cov}_s)]^{Y_3}[\pi((1-S_1)(1-S_2)+\mathrm{cov}_s)]^{Y_4}$$
$$[(1-\pi)((1-C_1)(1-C_2)+\mathrm{cov}_c)]^{U_1-Y_1}[(1-\pi)((1-C_1)C_2-\mathrm{cov}_c)]^{U_2-Y_2}[(1-\pi)(C_1(1-C_2)-\mathrm{cov}_c)]^{U_3-Y_3}[(1-\pi)(C_1C_2+\mathrm{cov}_c)]^{U_4-Y_4}$$

We used standard distributional families to represent our prior information. The choice of distributions discussed below is not unique and they may be replaced by other suitable densities, as needed. The prevalence is assumed to follow a beta prior distribution with

parameters α and β, π~beta(α_π, β_π). The sensitivities and specificities are also assumed to have beta prior densities such that S_j~beta(α_{Sj}, β_{Sj}), and C_j~beta(α_{Cj}, β_{Cj}), j=1,2. The feasible range of the covariance is determined by the sensitivities among the disease subjects and the specificities among the nondiseased subjects. The covariance parameters are taken to have uniform prior distribution, covs~uniform(0,min(S_1,S_2)-S_1S_2) and covc~uniform(0, min(C_1,C_2) -C_1C_2), where min(a,b) is the minimum of a and b. The Gibbs sampler can again be used to construct the marginal posterior densities of all parameters of interest. For two independence diagnostic tests, the full conditional distributions are as follows:

$$Y_1|U_1,\pi,S_1,C_1,S_1,C_1,\mathrm{cov}_s,\mathrm{cov}_c \sim Binomial(U_1,\frac{\pi\ (S_1S_2+\mathrm{cov}_s)}{\pi\ (S_1S_2+\mathrm{cov}_s)+(1-\pi)((1-C_1)(1-C_2)+\mathrm{cov}_c)}) \qquad \text{(app2.10)}$$

$$Y_2|U_2,\pi,S_1,C_1,S_1,C_1,\mathrm{cov}_s,\mathrm{cov}_c \sim Binomial(U_2,\frac{\pi\ (S_1(1-S_2)\ -\mathrm{cov}_s)}{\pi\ (S_1(1-S_2)\ -\mathrm{cov}_s)+(1-\pi)((1-C_1)C_2-\mathrm{cov}_c)}) \qquad \text{(app2.11)}$$

$$Y_3|U_3,\pi,S_1,C_1,S_1,C_1,\mathrm{cov}_s,\mathrm{cov}_c \sim Binomial(U_3,\frac{\pi\ ((1-S_1)\ S_2-\mathrm{cov}_s)}{\pi\ ((1-S_1)\ S_2-\mathrm{cov}_s)+(1-\pi)(C_1(1-C_2)-\mathrm{cov}_c)}) \qquad \text{(app2.12)}$$

$$Y_4|U_4,\pi,S_1,C_1,S_1,C_1,\mathrm{cov}_s,\mathrm{cov}_c \sim Binomial(U_4,\frac{\pi\ ((1-S_1)\ (1-S_2)\ +\mathrm{cov}_s)}{\pi\ ((1-S_1)\ (1-S_2)\ +\mathrm{cov}_s)+(1-\pi)(C_1C_2+\mathrm{cov}_c)}) \qquad \text{(app2.13)}$$

$$\pi\ |U_1,U_2,U_3,U_4,Y_1,Y_2,Y_3,Y_4,\alpha_\pi,\beta_\pi \sim Beta(Y_1+Y_2+Y_3+Y_4+\alpha_\pi,N\ -(Y_1+Y_2+Y_3+Y_4)+\beta_\pi) \qquad \text{(app2.14)}$$

$$S_1|Y_1,Y_2,Y_3,Y_4,\alpha_{S1},\beta_{S1} \sim Beta(Y_1+Y_2+\alpha_{S1},Y_3+Y_4+\beta_{S1}) \qquad \text{(app2.15)}$$

$$S_2|Y_1,Y_2,Y_3,Y_4,\alpha_{S2},\beta_{S2} \sim Beta(Y_1+Y_3+\alpha_{S2},Y_2+Y_4+\beta_{S2}) \qquad \text{(app2.16)}$$

$$C_1|U_1,U_2,U_3,U_4,Y_1,Y_2,Y_3,Y_4,\alpha_{C1},\beta_{C1} \sim Beta(U_3+U_4-(Y_3-Y_4)+\alpha_{C1},U_1+U_2-(Y_1+Y_2)+\beta_{C1}) \qquad \text{(app2.17)}$$

$$C_2|U_1,U_2,U_3,U_4,Y_1,Y_2,Y_3,Y_4,\alpha_{C2},\beta_{C2} \sim Beta(U_2+U_4-(Y_2-Y_4)+\alpha_{C2},U_1+U_3-(Y_1+Y_3)+\beta_{C2}) \qquad \text{(app2.18)}$$

$$\mathrm{cov}_s \sim uniform(0,\min(S_1,S_2)-S_1S_2) \qquad \text{(app2.19)}$$

$$\mathrm{cov}_c \sim uniform(0,\min(C_1,C_2)-C_1C_2) \qquad \text{(app2.20)}$$

Gibbs sampling is used to sample in turn from distribution app2.10 to distribution app2.20 in a similar fashion to the procedure used for the case of one diagnostic test outlined previously. The positive and negative predictive values for each cycle of the Gibbs algorithm

are again obtained directly from the relevant fractions of the true positive or negative subjects in each cell of the 2 by 2 table to the total observed number of subjects in that cell. Throughout, the Gibbs sampler was run for 100,000 cycles, the first 10,000 to assess convergence and the last 90,000 for inference. Each analysis was repeated from several different starting values, and convergence was assumed only if all runs provided very similar posterior distributions. Convergence of the algorithm here appeared to occur within the first 100-200 cycles, as evidenced by the monitoring of selected percentiles of the posterior samples. In general, the rate of convergence will depend on the starting values and the particulars of the data set and prior distributions. A computer program written in S-PLUS implementing all of the methods described in this paper is available from the first author (albert.tang@163.com).

6. WinBUGS program code for two independence diagnostic test

```
//
model;
{     pi ~ dbeta(api,bpi)
      sen1 ~ dbeta(as1,bs1)
      spe1 ~ dbeta(ac1,bc1)
      sen2 ~ dbeta(as2,bs2)
      spe2 ~ dbeta(ac2,bc2)

      api<-x[1]document+x[10]+x[11]+x[4]+api0
      bpi<-n-(x[1]+x[10]+x[11]+x[4])+bpi0

      as1<-x[1]+x[10]+as10
      bs1<-x[11]+x[4]+bs10
      ac1<-u[11]+u[4]-(x[11]+x[4])+ac10
      bc1<-u[1]+u[10]-(x[1]+x[10])+bc10

      as2<-x[1]+x[11]+ as20
      bs2<-x[10]+x[4]+ bs20
      ac2<-u[10]+u[4]-(x[10]+x[4])+ac20
      bc2<-u[1]+u[11]-(x[1]+x[11])+bc20

      p[1] <- pi * sen1 * sen2/(pi * sen1 * sen2 + (1 - pi) * (1 - spe1) * (1 - spe2))
      p[10] <- pi * sen1 * (1-sen2)/(pi * sen1 * (1-sen2) + (1 - pi) * (1 - spe1) * spe2)
      p[11] <- pi * (1-sen1) *sen2/(pi * (1-sen1) *sen2 + (1 - pi) *    spe1 * (1-spe2))
      p[4] <- pi * (1-sen1) * (1-sen2)/(pi * (1-sen1) * (1-sen2) + (1 - pi) * spe1 * spe2)

     for(i in 1:N)
      { x[i] ~ dbin(p[i],u[i])
      }
}
List(data format...)
//
```

Reference:

1. Branscum AJ, Gardner IA, Johnson WO (2005) Estimation of diagnostic-test sensitivity and specificity through Bayesian modeling. Preventive veterinary medicine 68: 145-163.
2. Spallone V, Ziegler D, Freeman R, Bernardi L, Frontoni S, et al. (2011) Cardiovascular autonomic neuropathy in diabetes: clinical impact, assessment, diagnosis, and management. Diabetes Metab Res Rev.
3. Pop-Busui R (2010) Cardiac autonomic neuropathy in diabetes: a clinical perspective. Diabetes care 33: 434-441.
4. Vinik AI, Ziegler D (2007) Diabetic cardiovascular autonomic neuropathy. Circulation 115: 387-397.
5. Straub RH, Lang B, Palitzsch KD, Scholmerich J (1997) Estimation of the cut-off value in cardiovascular autonomic nervous function tests: not-age-related criteria or the age-related 5th percentile. Journal of diabetes and its complications 11: 145-150.
6. Neil HA, Thompson AV, John S, McCarthy ST, Mann JI (1989) Diabetic autonomic neuropathy: the prevalence of impaired heart rate variability in a geographically defined population. Diabetic medicine : a journal of the British Diabetic Association 6: 20-24.
7. Risk M, Bril V, Broadbridge C, Cohen A (2001) Heart rate variability measurement in diabetic neuropathy: review of methods. Diabetes technology & therapeutics 3: 63-76.
8. Ziegler D, Dannehl K, Muhlen H, Spuler M, Gries FA (1992) Prevalence of cardiovascular autonomic dysfunction assessed by spectral analysis, vector analysis, and standard tests of heart rate variation and blood pressure responses at various stages of diabetic neuropathy. Diabetic medicine : a journal of the British Diabetic Association 9: 806-814.
9. Dendukuri N, Joseph L (2001) Bayesian approaches to modeling the conditional dependence between multiple diagnostic tests. Biometrics 57: 158-167.
10. Care A, Catalucci D, Felicetti F, Bonci D, Addario A, et al. (2007) MicroRNA-133 controls cardiac hypertrophy. Nat Med 13: 613-618.
11. Thayer JF, Merritt MM, Sollers JJ, 3rd, Zonderman AB, Evans MK, et al. (2003) Effect of angiotensin-converting enzyme insertion/deletion polymorphism DD genotype on high-frequency heart rate variability in African Americans. The American journal of cardiology 92: 1487-1490.
